# Potential of *Salvinia biloba* Raddi for removing atrazine and carbendazim from aquatic environments

**DOI:** 10.1007/s11356-022-23725-y

**Published:** 2022-10-25

**Authors:** Dana B. Loureiro, Luciana D. Lario, María S. Herrero, Lucas M. Salvatierra, Luís A. B. Novo, Leonardo M. Pérez

**Affiliations:** 1 Facultad de Química e Ingeniería del Rosario, Pontificia Universidad Católica Argentina (UCA-campus Rosario), Av. Pellegrini 3314, S2002QEO Rosario (Santa Fe), Argentina; 2 Instituto de Investigaciones en Ingeniería Ambiental, Química y Biotecnología Aplicada (INGEBIO), Facultad de Química e Ingeniería del Rosario, Pontificia Universidad Católica Argentina (UCA), Montevideo 3371, S2002QAC Rosario (Santa Fe), Argentina; 3 Consejo Nacional de Investigaciones Científicas y Técnicas (CONICET), Ministerio de Ciencia, Tecnología e Innovación Productiva, Godoy Cruz 2290, C1425FQB Buenos Aires, Argentina; 4 Scotland’s Rural College, West Mains Road, The King’s Buildings, Edinburgh, EH9 3JG UK; 5 Present Address: Laboratori de Microbiologia Sanitària i Mediambiental (MSMLab), Universitat Politècnica de Catalunya (UPC-BarcelonaTech), Rambla de Sant Nebridi 22, 08222 Terrassa (Barcelona), Spain

**Keywords:** Agrochemicals, Crop protection products, Phytoremediation, Biosorption, Carbendazim-resistant bacteria

## Abstract

In this exploratory study, naturally occurring *Salvinia biloba* Raddi specimens were assessed for atrazine and carbendazim polluted water remediation. Experiments were carried out over 21 days in glass vessels containing deionized water artificially contaminated with 0, 5, 10, and 20 mg L^−1^ of atrazine or carbendazim. Atrazine had a pronounced detrimental impact on *S. biloba*, as no biomass development was observed in all macrophytes exposed to this herbicide in the entire concentration range. However, carbendazim-treated plants were able to grow and survive in the polluted medium even when subjected to the highest concentration of this fungicide (i.e., 20 mg L^−1^). In addition, increased chlorosis and necrosis were also detected in plants subjected to carbendazim as a result of the high phytotoxicity caused by atrazine. A maximal removal efficiency of ~ 30% was observed for both pesticides at 5 mg L^−1^ and decreased with increasing concentrations of the pollutants. The spectrum of the FTIR-ATR analysis revealed the existence of various functional groups (e.g., amide, carboxyl, hydroxyl, phosphate, sulfate) on the plants, which could be related to pesticide biosorption. In addition, at the end of the 21-day assay, seven carbendazim-resistant bacteria could be isolated from the roots of fungicide-treated plants. Therefore, the use of autochthonous free-floating *S. biloba* macrophytes for phytoremediation of aquatic environments contaminated with carbendazim shows great promise. Still, additional research is required to further elucidate the plant-mediated carbendazim elimination process and the role of the herbicide-resistant bacteria, and seek alternative species capable of mitigating atrazine contamination.

## Introduction

Modern agriculture practices often lead to deleterious impacts on the surrounding ecosystems, among which pollution is perhaps the most concerning one. In particular, the widespread and excessive use of fertilizers and pesticides has resulted in the contamination of soils and natural water courses, mainly due to the transport of these compounds by surface runoff. Agrochemical dissemination is not only responsible for the death of aquatic organisms but also for harmful impacts across trophic levels, including human health (Nicolopoulou-Stamati et al. 2016; Nordgren and Charavaryamath 2018; Tudi et al. 2021).

Herbicides constitute nearly 60% of the pesticide volume utilized worldwide. While their global market size in 2021 was approximately $31.72 billion, by 2026 this value is forecasted to grow to $42.60 billion at a compound annual growth rate of 6.25% (Market Data Forecast 2022). Among herbicides, atrazine (2-chloro-4-ethylamino-6-isopropylamino-*s*-triazin) has been receiving increasing attention as one of the most broadly employed pesticides, only trailing after glyphosate (Dayan 2019; Sharma et al. 2019). Atrazine is part of the chlorinated triazine herbicide group, which exhibit moderate mobility and high persistence in soil and water. It is mostly employed as a selective herbicide to avert the emergence of grassy and broadleaf weeds in a wide range of crops (e.g., corn, sugarcane, sorghum, cotton, among others) and turf (Cheremisinoff and Rosenfeld, 2011). Major concerns have been raised about the use of atrazine following reports about its slow degradability, which results in significant migration as runoff to surface waterbodies and groundwater alike. Yet, because of its displacement from soil, the levels of atrazine in surface water are greater in edge-of-field runoff, where concentrations reach low milligrams per liter at the beginning of major rainfall events following application (de Albuquerque et al. 2020). Moreover, contamination of tap water by atrazine in areas where it is applied have been reported (Rohr 2021). As a potent endocrine disruptor, atrazine has a sublethal impact on a wide array of aquatic organisms and poses severe risks on human development and reproduction, including cancer induction (Boffetta et al. 2013; Stayner et al. 2017; Singh et al. 2018; Sharma et al. 2019). Consequently, atrazine has been banned by the European Union (EU) since 2004, and in the USA, a limit of 3 μg L^−1^ in drinking water has been established by the Environmental Protection Agency (USEPA) and a health guideline limit of 0.1 μg L^−1^ in tap water has been recommended by the Environmental Working Group (EWG 2021a). Analogously, the World Health Organization (WHO) suggests a guideline value of 2 μg L^−1^ for drinking water (Wirbisky and Freeman 2015). Still, in the USA, atrazine concentrations in tap water exceeded the EWG’s Health Guideline value in 24 states (EWG 2021b). A recent assessment of drinking water from rural agricultural areas in Nigeria detected atrazine levels ranging from 10 to 80 μg L^−1^ (Owagboriaye et al. 2022).

On the other hand, carbendazim (2-methoxy-carbamoyl benzimidazole) is a broad-spectrum systemic fungicide that is commonly applied on several crops including tobacco, fruits, vegetables, and cereals (Sharma et al. 2019). This pesticide has been included in the EU’s list of priority endocrine-disrupting agents (Singh et al. 2016) and is currently classified as a mutagenic substance by the same governing body (ECHA 2022). The widespread use of carbendazim (often over the recommended dosage), added to the fact that this fungicide shows low degradability, has led to its persistence in the environment (Dong et al. 2018). Although the toxic effects of carbendazim are well known since the early 1980s, increasing concerns about its action as environmental endocrine disruptor have made it a hot topic (Singh et al. 2016; Madboli and Seif 2021). Some studies have revealed reproductive disturbances, toxicity, and mutagenicity in model organisms exposed to carbendazim (Li et al. 2020), as well as carbendazim-induced adverse effects on the liver, kidney, and endocrine glands, and their hormonal levels (Rama et al. 2014). Thus, carbendazim has been barred in the UK and USA (Singh et al. 2016; Li et al. 2020), while the EU has established maximum limits on the amount of carbendazim residues present in foodstuff (Commission Directive 98/82/EC). However, this fungicide is one of the most used pesticides worldwide, behind glyphosate, atrazine, chlorpyrifos, and endosulfan (Sharma et al. 2019). In fact, the use and production of carbendazim is still allowed in most developing countries like China, South Africa, Brazil, Argentina, and India. Therefore, the search for new tools to mitigate the eco-damage and health-associated risk triggered by the occurrence of pesticides in the environment is of great interest and growing demand.

The contamination of aquifers by atrazine and carbendazim poses major environmental problems since under natural environmental conditions both compounds are very stable, and concentrations at parts per million level (ppm; mg L^−1^) of both pesticides have been identified in ground and surface waters (Singh et al. 2016; de Albuquerque et al. 2020). In Argentina, water courses adjacent to agricultural areas have been proved to contain quantities of pesticides harmful to health (De Gerónimo et al. 2014; Pérez et al. 2017; Corcoran et al. 2020). Hence, bioremediation methods for the eco-management of pesticide-containing wastewaters are needed as environment-friendly and inexpensive reclamation approaches, especially in developing countries where regulations are lagging behind and conventional cleanup solutions are prohibitive. Phytoremediation has been extensively implemented for treating pesticides in wetlands systems using different helophyte species (i.e., emergent plants) (Matamoros et al. 2012; Vymazal and Březinová 2015; Vryzas 2016). Yet, an alternative approach based on aquatic free-floating plants can be utilized for agrochemical remediation or biomonitoring in water bodies (Guimarães et al. 2011; Della Vechia et al. 2016; Escolá Casas and Matamoros 2022). In this context, two subsets of phytoremediation are particularly promising for alleviating the water contamination by crop protection products: rhizodegradation and phytodegradation. While the former relies on the activity of rhizospheric bacteria activity for degrading organic pollutants, the latter is grounded on the enzymatic degradation of organic pollutants in planta (Bernardino et al. 2020). For instance, the genus *Salvinia* has exhibited great promise due to elevated growth rate and impressive capability to survive under unfavorable environmental conditions (Dhir 2009). *Salvinia* comprises around 20 species located in temperate and tropical zones across the planet, from which the most representative are *Salvinia auriculata* Aubl., *Salvinia minima* Baker, *Salvinia natans* (L.) all., and *S. biloba*; however, there are no studies on *S. biloba* regarding the elimination of toxic pesticides from aquatic systems (Mustafa and Hayder 2021). Therefore, the objective of this exploratory work was to assess the potential of free-floating *S. biloba* for removing atrazine and carbendazim from water in order to determine its suitability for relevant phytoremediation programs.

## Materials and methods

### Plant collection and characterization

**Fig. 1:**
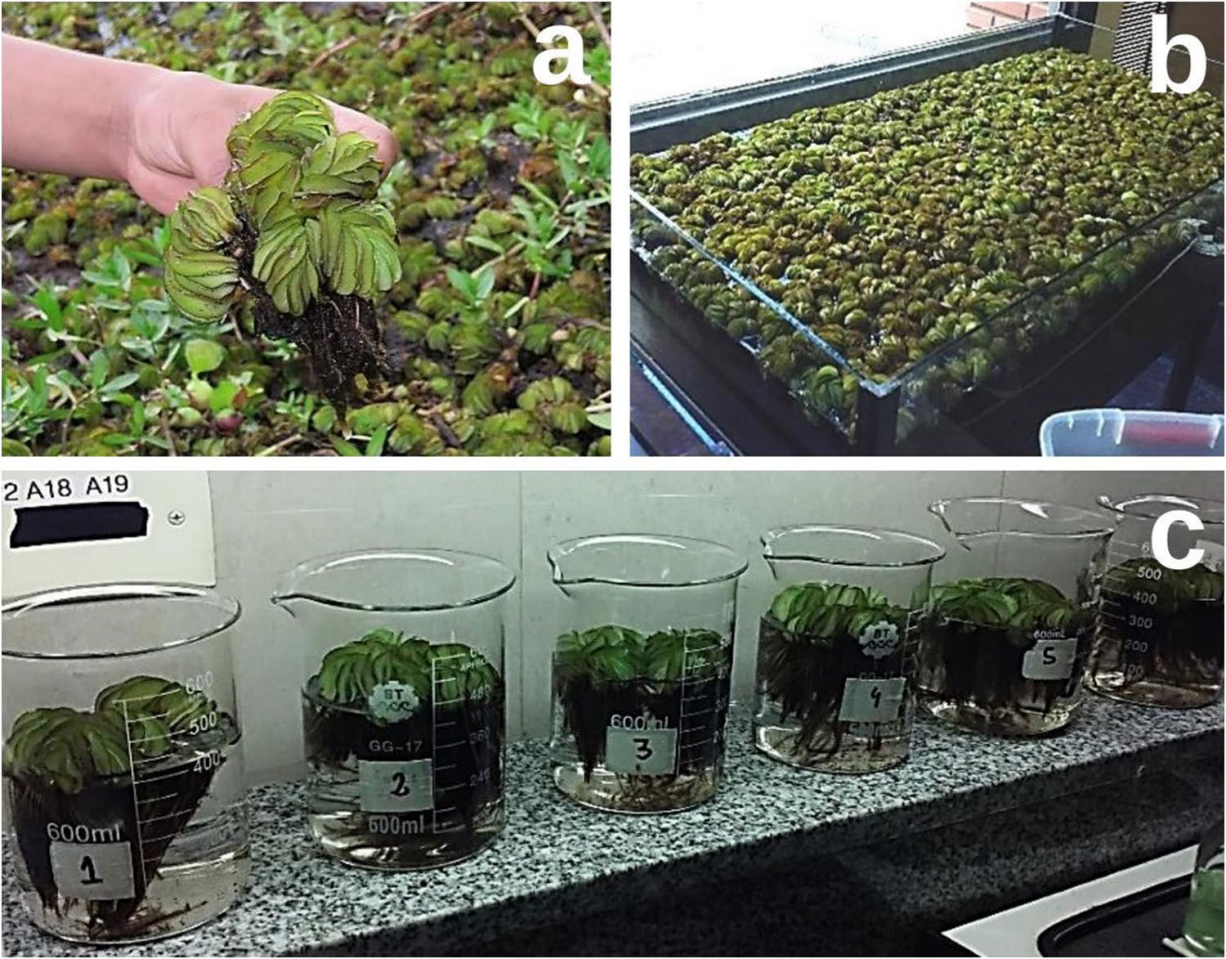
**a** Naturally occurring *S. biloba* free-floating specimen collected from a floodplain located at the Middle Paraná River (Entre Ríos, Argentina) as described in the text. **b** Glass aquaria for macrophyte conservation and acclimation. **c** Representative photograph of the batch study conducted for 21 days under laboratory-controlled conditions using fresh plant biomass (20 g) in 400-mL experimental units

The Paraná River’s adjoining ecosystem is characterized by a wide wetland area featuring rich and varied aquatic vegetation (Ferreira et al. 2011). In this study, naturally occurring *Salvinia* sp. were carefully collected (Fig. 1a) from a low-depth lagoon situated at the coordinates 32°52′35″S and 60°40′33″W (Entre Ríos, Argentina). This location corresponds to a natural wetland area at the Middle Paraná River already described by our group in previous works (Tello Zevallos et al. 2018; Castillo Loría et al. 2019; Emiliani et al. 2020, 2021). During collection, macrophytes were kept at ambient temperature in plastic containers filled with water from the river. Afterwards, the plants were transferred to the laboratory and developed hydroponically in glass tanks (20 L) containing a combination of tap and lagoon water (1:1, *v/v*) at room temperature (24 ± 2 °C) (Fig. 1c). The specimens of *Salvinia* sp. were categorized as *S. biloba* based on morphological characteristics according to Castillo Loría et al. (2019).

### Plant exposure to pesticides

Selected *S. biloba* plants (20 g wet basis) were placed in beakers (600 mL) filled with 400 mL of a pure water solution artificially contaminated with 0 (control), 5, 10, and 20 mg L^−1^ of atrazine or carbendazim previously prepared in methanol (100 mg L^−1^ pesticide stock solution) (Fig. 1c). The control was also prepared using the methanol stock solution (final methanol concentration of 2%) to eliminate bias. No additional nutrients were supplied to the medium. Two experimental units (*n* = 2) were utilized as replicates for each pesticide concentration. The experiments were carried out over 20 days at 24 ± 2 °C employing artificial lighting (photosynthetic Photon Flux Density of 50 µmol m^−2^ s^−1^; Osram Dulux L HE, Germany) with 12-h photoperiods (Castillo Loría et al. 2019; Emiliani et al. 2020). Photographic records of the plant material were made during the entire experiment to visually evaluate phenotypic alterations to *S. biloba* biomass associated to pesticide toxicity. At the end of the trials, samples of the water column were collected to verify residual atrazine and carbendazim concentration by high-performance liquid chromatography (HPLC). In addition, total biomass was collected and dried until constant weight at 90 °C for Fourier-Transform Infrared-Attenuated Total Reflectance (FTIR-ATR) analysis.

### Chlorosis assessment

The extent of chlorosis in the *S. biloba* specimens at the end of the experiment was determined with the aid of the image analysis software Fiji (Schindelin et al, 2012).

### Pesticide quantification

Residual atrazine and carbendazim concentrations were quantified from a 10-mL supernatant of centrifuged (5000 × *g*, 5 min) water samples using an Agilent Technologies 1100 Series HPLC device (Agilent Technologies, Santa Clara, CA, USA) outfitted with an analytical ZORBAX Eclipse XDB-C18 reversed-phase column (150 × 4.6 mm, particle size 5 μm; Agilent Technologies). An optimal elution solution composed by methanol/water (55:45 *v*/*v*) was applied over 10 min at a flow rate of 1 mL min^−1^, while the column temperature was kept at 40 °C. The signal was measured using an UV–vis detector set at a wavelength of 254 nm. For calibration, standard solutions of atrazine and carbendazim (0.3, 0.6, 1.0, 2.0, 2.5, and 3.0 mg L^−1^) were made through the dilution of a 100 mg L^−1^ stock solution with the required volume of pure water in calibrated glassware. The calibration processes displayed reproducible linear relationships (*R*^2^ > 0.995)*.*

### FTIR-ATR analysis

The spectra of dry *S. biloba* biomass were obtained using an FTIR spectrophotometer (Shimadzu IR Prestige-21, Tokyo, Japan) equipped with ATR accessory. The readings were recorded in the scanning range of 500–4000 cm^–1^. In total, 20 scans were carried out at a resolution of 2 cm^–1^ (Tello Zevallos et al. 2018).

### Bacterial isolation from carbendazim-treated macrophytes

Potentially carbendazim-resistant bacteria were isolated from the roots of *S. biloba* specimens after 21 days of pesticide exposure. Plant samples were gently collected and submerged in 50 mL of sterile phosphate buffer (PBS, pH 7.4) and the supernatants were serially diluted from 10^–1^ to 10^–5^. In addition, the root’s surface was disinfected with 70% ethanol during 1 min and rinsed with sterile distilled water. Then, roots were cut into pieces and 1.0 g of each one was poured in PBS. Serial dilutions were made and aliquots of 0.1–0.01 mL from dilution 10^–3^ to 10^–6^ were plated in Petri dishes containing bacterial Tryptic Soy agar (Britania S.A., Buenos Aires, Argentina). After being incubated for 48 h at 37 °C, colonies were sub-cultured in Tryptic Soy agar broth (TSB, Britania S.A.) and Gram stained for further characterization using commercially available biochemical tests (RapID NF Plus System; Remel, UK). The isolates were maintained in TSB medium with 20% glycerol on Eppendorf tubes at − 70 °C.

### Microbial growth estimation in liquid culture

The growth of the isolated microorganisms in liquid medium supplemented with carbendazim was assessed by a modification of the method describe by Pérez et al. (2014). In short, overnight cultures of the bacterial strains isolated from the roots of carbendazim-treated *S. biloba* plants were adjusted to McFarland 0.5 standard through the addition of saline solution (~ 1.5 × 10^8^ cfu mL^−1^) and treated as test inoculum. One milliliter of the latter was added to 1 mL of a pesticide-containing solution prepared in TSB (pH = 7.4.) (Britania S.A.) and thoroughly mixed by vortexing for 10 s. The carbendazim concentrations tested were 0, 5, 10, and 20 mg L^−1^. A control tube solely containing broth medium was assessed to discard potential contaminations. All the tubes were incubated overnight (16–18 h) at 37 °C in a culture chamber. The growth of the microorganisms (i.e., turbidity) in the test tubes was distinguished by the unaided eye and photographically recorded.

## Results and discussion

### Efficiency of pesticide removal by *S. biloba*

**Fig. 2:**
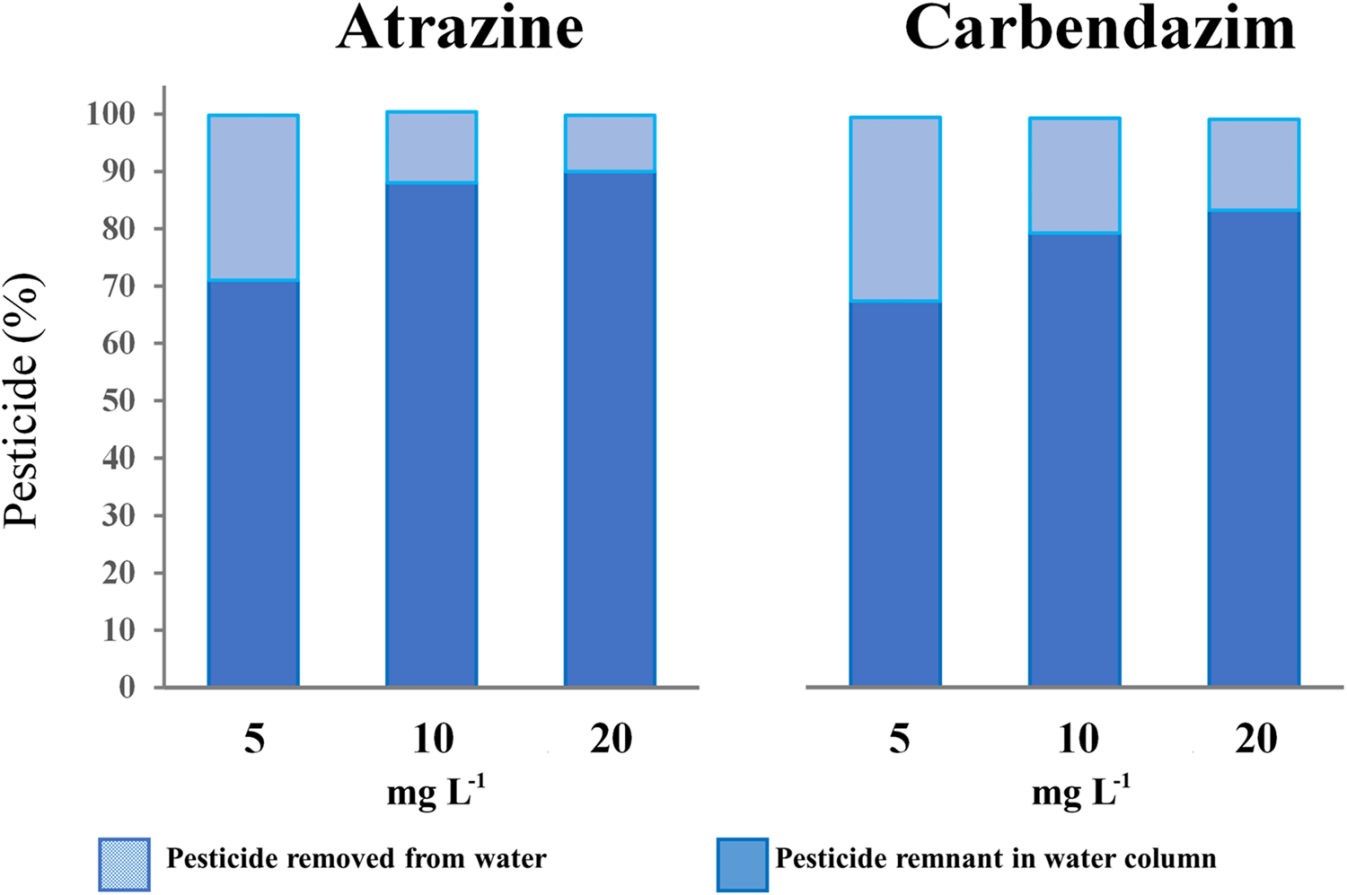
Percentage (%) of atrazine and carbendazim removal from water column by naturally occurring *S. biloba* specimens after 21-day exposure to 5, 10, or 20 mg L^−1^ of the pesticides in independent experiments

Overall, *S. biloba* showed limited capacity for removing atrazine and carbendazim from artificially contaminated water samples at the concentration range tested in this study. As depicted in Fig. 2, at 5 mg L^−1^ of atrazine or carbendazim, the total amount of pollutant removed by the plants was ~ 30% for both pesticides. Moreover, increasing pesticide concentrations lessened the removal efficiency by the macrophytes. These results are in tune with those described by Guimarães et al. (2011) who noted that the percentage of atrazine absorbed by three related macrophytes species (*Azolla caroliniana* Willd., *Lemna gibba* L., and *S. minima*) decreased when the atrazine concentration in the water samples increased from 0.1 to 10 mg L^−1^. However, in our study, *S. biloba* proved to be a little more efficient in removing the fungicide than the herbicide at 10 and 20 mg L^−1^. For example, the percentage of atrazine removed from the water column shifted from 12 to 10% when the herbicide levels increased from 10 to 20 mg L^−1^, while the amount of carbendazim eliminated from water varied from 20 to 16% when doubling the fungicide concentration from 10 to 20 mg L^−1^.

Accordingly, positive results have been reported for plants with prominent phytoremediation potential that thrive in environments highly polluted with pesticides. Hence, it has been suggested that some of these plants exhibited pesticide detoxification mechanisms involving the action of glutathione, through the enzymatic activity of glutathione-*S*-transferase (GST). For example, Marcacci et al. (2006) informed about the ability of *Chrysopogon zizanioides* (L.) Roberty for removing atrazine following a hydroponic study. Moreover, these authors noted the role of glutathione in the atrazine detoxification process. Recently, dos Santos et al. (2021) have demonstrated that macrophytes exhibiting greater GST activity also presented enhanced biotransformation potential against three different biocides (chlorothalonil, 4,5-dichloro-*N*-octyl-3(2H)-isothiazolone, and dichlofluanid). Furthermore, the free-floating macrophyte *L. gibba* also possesses similar detoxifying mechanisms involving glutathione (Alkimin et al. 2020). Moreover, some *Lemnaceae* species have been considered to be tolerant to triazinic herbicides, such as atrazine (Kumar and Han 2010). Therefore, it is possible that similar tolerance mechanisms may be shared among evolutionarily related macrophyte species.

Our results showed that *S. biloba*’s phytoremediation efficiency decreased with increasing pesticide concentration in the solution (Fig. 2), suggesting that this autochthonous aquatic fern is better suited for the clean-up of water contaminated with relatively low levels of pesticides. Thus, these findings are very promising because when field run-off is diluted upon entering a stream or lake, the resulting atrazine concentrations are generally much lower than those evaluated at the present study (de Albuquerque et al. 2020).

**Fig. 3:**
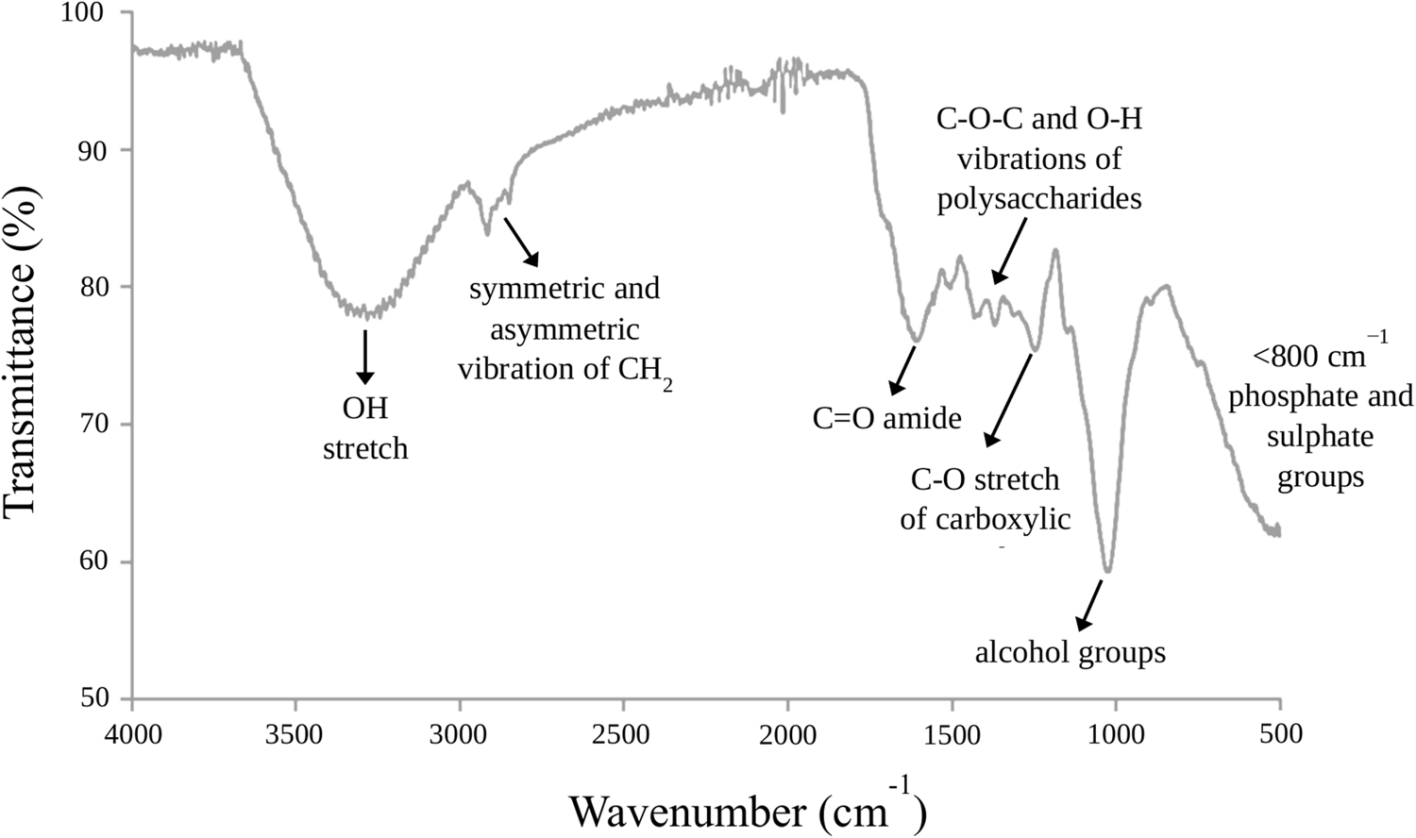
FTIR-ATR spectrum of *S. biloba* biomass

The principal retention phenomena governing the migration, transformation, and environmental effects of pesticides are adsorption and desorption. The adsorption and mobility of organic pesticides rest on the molecule’s ionic or neutral character, on its solubility in water, polarity, and the number of binding sites of the adsorbent organic matter (Cederlund et al. 2016). In this view, *Salvinia* biomass exhibits a large specific surface area with abundant macromolecules (i.e., carbohydrates, lipids, and proteins), which may explain the pesticide-plant binding mechanism (Dhir 2009; Tello Zevallos et al. 2018). As shown in Fig. 3, the FTIR-ATR spectrum displayed the presence of carboxyl, phosphate, amide, hydroxyl, thiol, and other functional groups at the surface of the plant that could mediate pesticide biosorption via several types of interactions, like π–π interactions, acid–base behavior, electrostatic attractions, hydrogen bonding, and ion-exchange properties. The bands at 3400–3250 cm^−1^ are allocated to the –OH stretch of polymeric compounds; the band at 2940–2830 cm^–1^ refers to the asymmetric and symmetric vibration of methylene (CH_2_), respectively. The C=O stretch of amide corresponds to the peak found at 1630 cm^–1^, while at 1130–1000 cm^−1^ the vibration of C–O–C and O–H of polysaccharides was detected. The distinct peaks at 1034–1036 cm^–1^ are related to alcohol groups, and the 1250 cm^–1^ peak is the C–O stretch of carboxylic acids. The bands below 800 cm^−1^ represent the fingerprint zone that includes phosphate- and sulfur-containing functional groups (Tello Zevallos et al. 2018). Therefore, it is expected that both physical adsorption and chemisorption can contribute to the retention of atrazine and carbendazim by *S. biloba*. In fact, considering that the whole abaxial surface of the leaf was in contact with water, it is highly possible that pesticide removal from aquatic environments may involve two different biosorption mechanisms: absorption by the submerged roots and also a direct absorption by leaves contacted to water (Guimarães et al. 2011).

### Phenotypic evaluation of pesticide phytotoxicity in *S. biloba*

**Fig. 4:**
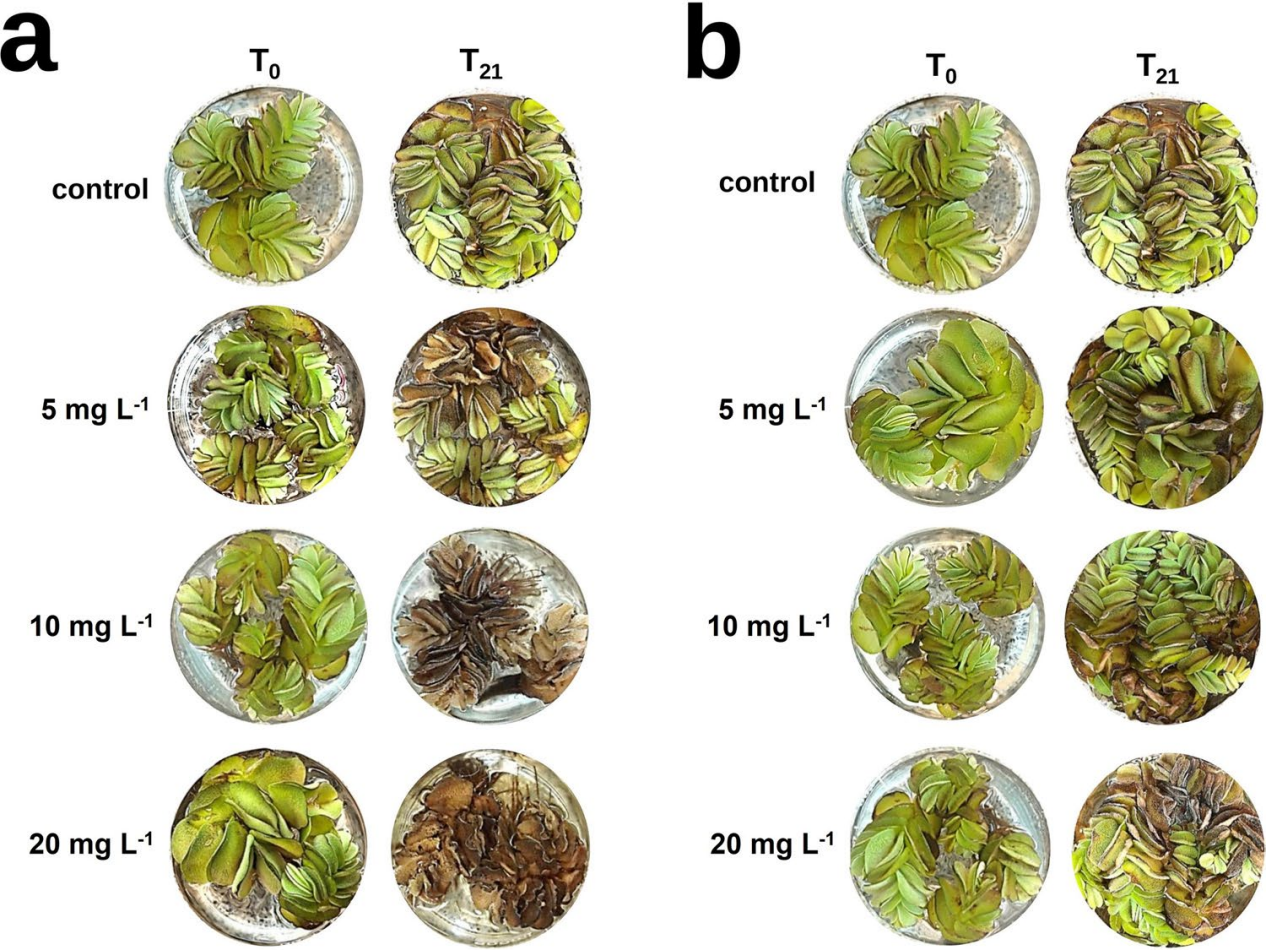
Photographic records displaying phenotypic alterations in *S. biloba* specimens after 21-day exposure to water contaminated with 0 (control), 5, 10, or 20 mg L^−1^ of atrazine (**a**) and carbendazim (**b**) compared to control plants

**Fig. 5:**
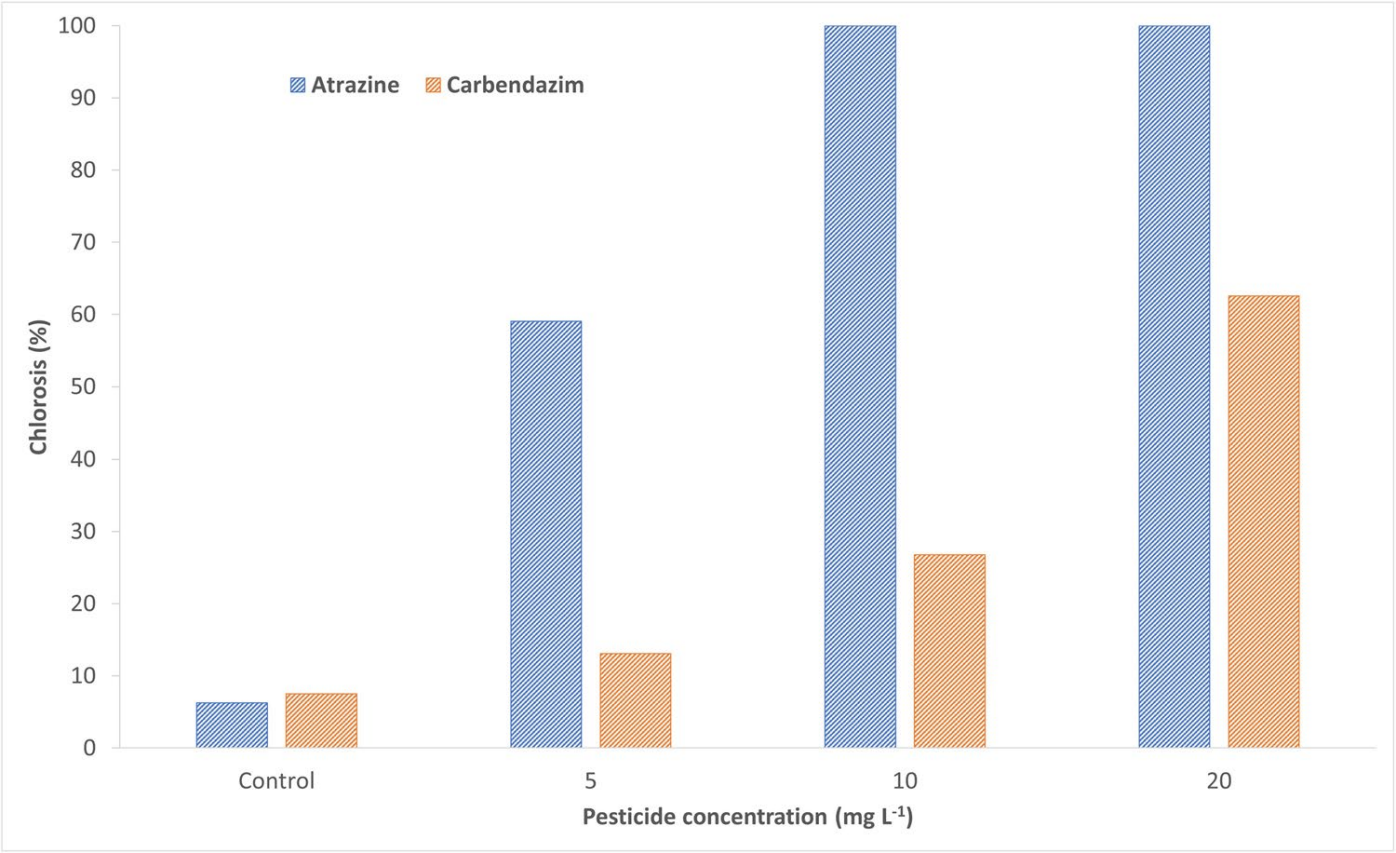
Chlorosis percentage in *S. biloba* specimens after 21-day exposure to water contaminated with 0 (control), 5, 10, or 20 mg L^−1^ of atrazine and carbendazim

A photographic record was made on *S. biloba* specimens exposed to different atrazine and carbendazim concentrations. As can be appraised in Fig. 4a, plants exhibited a marked decay caused by atrazine exposure. In general, increased leaf chlorosis, leaf turgor loss (size and shape changes), and evidence of necrosis (cell death) were detected across juvenile and developed fronds. In addition, a continuing adverse reaction of *S. biloba* to atrazine was clearly perceived during the exposure time. The external morphological harm, like the incidence of necrotized areas on the leaves, was intensified with increasing herbicide concentrations in the growing medium. Moreover, growth was hampered in plants exposed to all three concentrations of atrazine. These results were somewhat expected since atrazine is a photosynthesis disrupter (Zhu et al. 2009). It has been reported that this herbicide induces severe damage on photosystems I and II by blocking the electron transport, which leads to a marked reduction in photosynthetic oxygen production and affects carbon assimilation in the target plants (Mohammad et al. 2010). However, some plants exhibit tolerance to atrazine, suggesting the existence of underlying mechanisms that could be advantageous for phytoremediation programs (Guimarães et al. 2011). Hindered photosynthesis can be identified by the occurrence of chlorosis and necrosis and stunted growth. These traits were observed and quantified in *S. biloba* plants exposed to atrazine during our study (Figs. 4a and 5, respectively). Based on our observations, it is likely that the naturally occurring *S. biloba* specimens used in this study did not develop the mechanisms to tolerate and attenuate the effects of atrazine at the selected concentrations, suggesting a low efficiency in alleviating environments contaminated by this herbicide at the parts-per-million (ppm) levels. Indeed, at the end of the experiment, the percentage of chlorosis for plants subjected to 5 mg L^−1^ was 59%, whereas those exposed to 10 and 20 mg L^−1^ were completely necrotized (Figs. 4a and 5).

On the other hand, carbendazim-exposed *S. biloba* plants showed a remarkably smaller degree of decline, especially at 5 and 10 mg L^−1^ of carbendazim with relative chlorosis percentages of 13 and 27%, respectively (Figs. 4b and 5). Noticeably, the plants were able to grow and develop at the three tested concentrations of the fungicide in water. However, leaf chlorosis and necrosis were pronounced in *S. biloba* plants exposed to 20 mg L^−1^ of carbendazim. Numerous plants have the ability to absorb and metabolize organic compounds or release exudates that foster the growth of beneficial rhizospheric microbes that further degrade or complex the pollutants (Guimarães et al. 2011; Escolá Casas and Matamoros 2022). Indeed, recent studies with aquatic floating plants including *Lemma* sp. and *Salvinia* sp. suggest that root exudates promote microbe biodegradation of organic pollutants like diclofenac or naproxen (Matamoros et al. 2012; Fester et al. 2014; Garcia-Rodríguez et al. 2015; Escolá Casas and Matamoros 2022). In a more recent work, Paz et al. (2019) noted a change in the root exudation pattern of *Phragmites australis* (Cav.) Trin. ex Steud. when exposed to micropollutants. Similarly, Jin et al. (2019) demonstrated that exudates from maize seedlings stimulate bacterial alleviation of phenol in water. Also, a bench-scale constructed wetland study conducted by Sauvêtre et al. (2020) showed that exposition to sulfamethoxazole and diclofenac induced significant changes in the rhizospheric bacterial community, causing an increase in microorganisms with enhanced biodegradation and plant growth–promoting traits (Sauvêtre et al. 2020). Although the biodegradation of pesticides and other organic pollutants is primarily microbe driven, the influence of root exudation on the overall efficiency of the process is evident (Segura and Ramos 2013).

Due to the slow rates of degradation of carbendazim by physical and abiotic processes, microbe-mediated mechanisms constitute the most important degradative pathway for this compound (Singh et al. 2016). Several bacterial strains have revealed potential to degrade carbendazim including members of the genera *Azospirillum*, *Aeromonas*, *Alternaria*, *Bacillus*, *Brevibacillus*, *Nocardioides*, *Pseudomonas*, *Ralstonia*, *Rhodococcus*, *Sphingomonas*, *Streptomyces*, and *Trichoderma* (Xinjian et al. 2013; Singh et al. 2016; Panda et al. 2017).

**Fig. 6:**
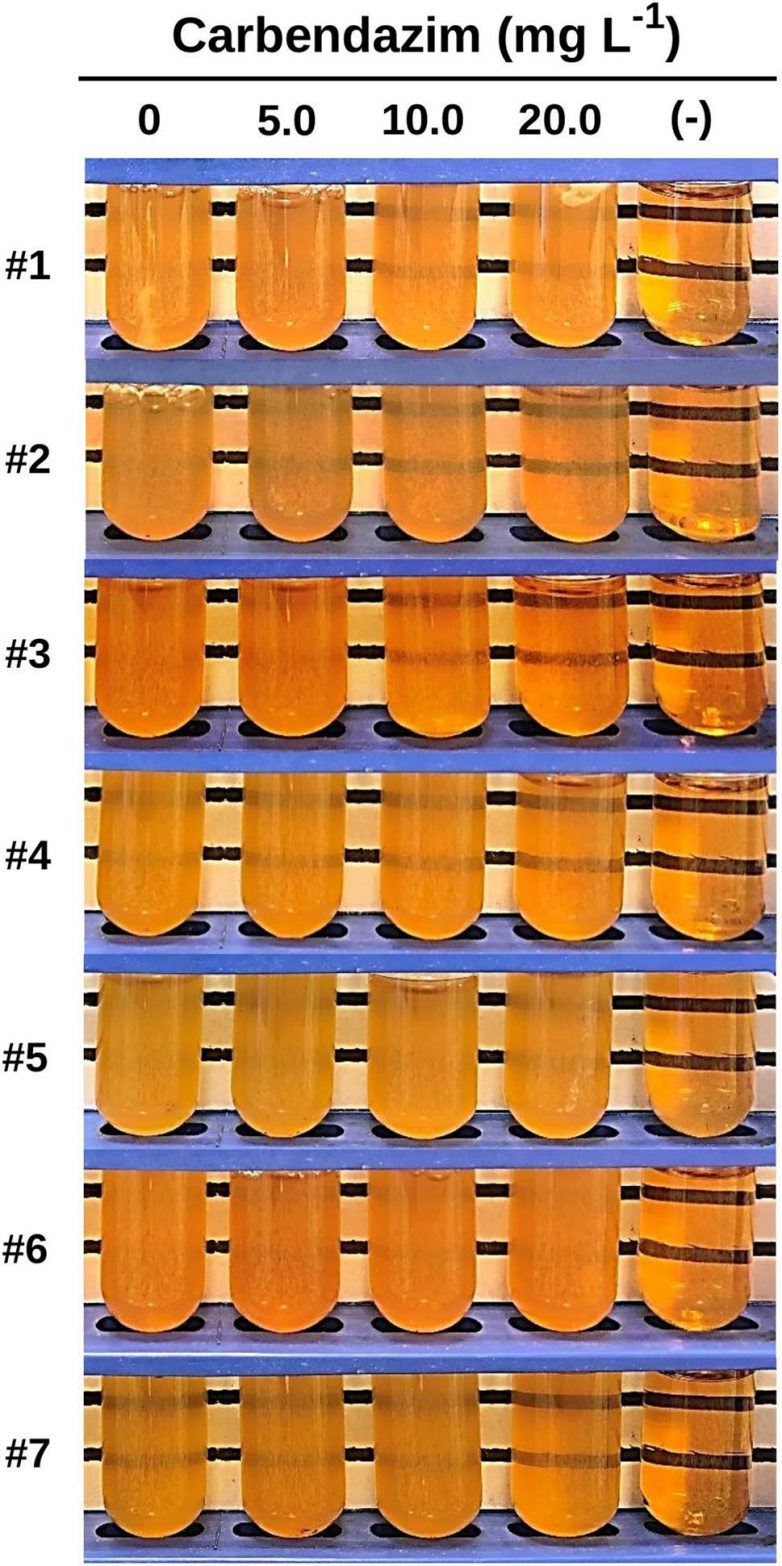
Representative photographs showing microbial growth (cloudy tubes) of seven carbendazim-resistant bacteria isolated from *S. biloba* roots after 21-day pesticide exposure. All strains were able to survive and reproduce in culture broth (TSB) contaminated with 5 to 20 mg L^−1^ carbendazim after 24-h incubation at 37 °C. (–), test tube with no bacteria inoculated

In this work, we were able to isolate seven carbendazim-resistant bacteria from the roots of *S. biloba* after 21-day fungicide exposure (Fig. 6). Therefore, it seems possible that some endophytic bacteria could be involved in the carbendazim resistance observed for the naturally occurring macrophytes used in this study. A preliminary identification of the isolated microorganisms was performed using Gram-staining and biochemical tests (RapID NF Plus System). Using these approaches, the identification of the isolated strains was unclear or not firmly conclusive (identity < 90%). This may be related to the fact that the RapID method is designed on the basis of basic biochemical characteristics for detecting most enterobacteria, and its use on environmental microbes may not consider the high unpredictability of wild strains, where the lack of reference data for most biochemical assays could influence the accuracy of the result. Even so, five strains were positively identified as Gram-negative bacilli and two round-shape Gram-positive bacteria. Therefore, the results of microbial growth on selective-differentiating, chromogenic media should be regarded illustrative and useful for picking the adequate conditions for further analysis, as well as for choosing the tools for future studies using DNA sequencing techniques.

In conclusion, further research is needed to clarify the role of bacteria associated with naturally occurring *S. biloba* in phytoremediation of carbendazim-polluted water contaminated since highly efficient and ecologically competitive microbes are required to remediate pesticide-contaminated environments. Nevertheless, bacterial biodegradation shows great promise as an environment-friendly and cost-effective strategy for bioaugmented phytoremediation of water reservoirs contaminated by agrochemicals.

## Conclusions

The potential of native free-floating *S. biloba* for phytoremediation of atrazine and carbendazim was assessed as an alternative to traditional cleaning methods for pesticide removal from water environments. Decreased biomass growth and severe leaf necrosis were observed in all plants exposed for 21 days to atrazine. However, *S. biloba* proved to be highly tolerant to carbendazim, and this behavior seems to be related with the occurrence of endophytic bacteria in the plant’s roots, which displayed resistance to the fungicide in growth assays performed at liquid medium. Overall, *S. biloba* showed relatively low potential for the removal of atrazine and carbendazim at the concentrations tested (i.e., 5, 10, and 20 mg L^−1^). Furthermore, the percentage of pesticides eliminated from the water decreased with increasing pollutant concentration in the solution. This fact could be related with a limited availability of the functional groups located at the plant surface involved in pesticide biosorption—described from the FTIR-ATR analysis—since pollutant concentrations used at this work were significant. Yet, it should be noted that typical concentrations of atrazine and carbendazim in natural water bodies are smaller than those employed in this study. Thus, the application of these floating macrophytes to alleviate pesticide contamination in aquatic environments appears to be conditioned by their concentration. However, although further research is needed, the use of microbial bioaugmented phytoremediation could be an attractive approach for enhancing the effectiveness of the bioprocess. Important follow-up research may encompass the assessment *S. biloba*’s capacity to alleviate water contamination by other agrochemicals, in-depth analysis of rhizospheric microbe activity (including isolation, identification, and characterization of relevant microorganisms), and detailed study of the underlying degradation mechanisms.

## Data Availability

The datasets used and/or analyzed during the current study are available from the corresponding author on reasonable request.
